# Intravenous lipid emulsion for local anaesthetic systemic toxicity in pregnant women: a scoping review

**DOI:** 10.1186/s12884-024-06309-1

**Published:** 2024-02-14

**Authors:** Makoto Tsuji, Masafumi Nii, Marie Furuta, Shinji Baba, Takahide Maenaka, Shigetaka Matsunaga, Hiroaki Tanaka, Atsushi Sakurai, on behalf of the Japan Resuscitation Council (JRC) of the Guideline Editorial Committee, Rie Kato, Jun Takeda, Masahiro Nakao, Eishin Nakamura, Tomoyuki Yamashita, Yoshinori Yamahata, Naosuke Enomoto, Shinji Baba, Yuki Hosokawa

**Affiliations:** 1 Department of Obstetrics and Gynecology, Saiseikai Mastusaka General Hospital, Mastusaka, Mie Japan; 2 Department of Obstetrics and Gynecology, Mie University School of Medicine, Tsu, Mie Japan; 3 Department of Human Health Sciences, Graduate School of Medicine, Kyoto University, Kyoto, Kyoto Japan; 4 Department of Obstetrics and Gynecology, Tokyo Metropolitan Tama Medical Center, Fuchu, Tokyo, Japan; 5 Regional Medical Care Planning Division, Health Policy Bureau, Ministry of Health, Labour and Welfare, Tokyo, Japan; 6 Department of Obstetrics and Gynecology, Saitama Medical Center, Saitama Medical University, Kawagoe, Saitama Japan; 7 Department of Acute Medicine, Division of Emergency and Critical Care Medicine, Nihon University School of Medicine, Itabashi, Tokyo, Japan; 8 Japan Resuscitation Council, Shinjuku, Tokyo, Japan

**Keywords:** Lipid emulsion, Emergency medicine, Anaesthesia, Analgesia – Obstetric, Shock, Resuscitation

## Abstract

**Background:**

Local anaesthetic systemic toxicity (LAST) is a rare but life-threatening complication that can occur after local anaesthetic administration. Various clinical guidelines recommend an intravenous lipid emulsion as a treatment for local anaesthetic–induced cardiac arrest. However, its therapeutic application in pregnant patients has not yet been established. This scoping review aims to systematically identify and map the evidence on the efficacy and safety of intravenous lipid emulsion for treating LAST during pregnancy.

**Method:**

We searched electronic databases (Medline, Embase and Cochrane Central Register Controlled Trials) and a clinical registry (lipidrescue.org) from inception to Sep 30, 2022. No restriction was placed on the year of publication or the language. We included any study design containing primary data on obstetric patients with signs and symptoms of LAST.

**Results:**

After eliminating duplicates, we screened 8,370 titles and abstracts, retrieving 41 full-text articles. We identified 22 women who developed LAST during pregnancy and childbirth, all presented as case reports or series. The most frequent causes of LAST were drug overdose and intravascular migration of the epidural catheter followed by wrong-route drug errors (i.e. intravenous anaesthetic administration). Of the 15 women who received lipid emulsions, all survived and none sustained lasting neurological or cardiovascular damage related to LAST. No adverse events or side effects following intravenous lipid emulsion administration were reported in mothers or neonates. Five of the seven women who did not receive lipid emulsions survived; however, the other two died.

**Conclusion:**

Studies on the efficacy and safety of lipids in pregnancy are scarce. Further studies with appropriate comparison groups are needed to provide more robust evidence. It will also be necessary to accumulate data—including adverse events—to enable clinicians to conduct risk–benefit analyses of lipids and to facilitate evidence-based decision-making for clinical practice.

**Supplementary Information:**

The online version contains supplementary material available at 10.1186/s12884-024-06309-1.

## Introduction

Local anaesthetic systemic toxicity (LAST) is a rare but potentially life-threatening side effect associated with the administration of local anaesthetics. LAST occurs when blood concentrations of local anaesthetics reach a toxic range, either by direct arterial or intravenous administration or by gradual absorption from extravascular tissue [1]. The incidence of LAST is estimated to be up to 1 in 500 peripheral nerve blocks and may occur in up to 4 in 10,000 epidural procedures [2, 3]. Although prevention is the most important element, LAST can still occur despite best clinical practices [4]. For appropriate management, early recognition of LAST signs and symptoms is essential.

LAST has two clinical manifestations: central nervous system toxicity and cardiovascular toxicity [4]. Central nervous system toxicity is classically biphasic, including an initial excitatory phase (e.g. dizziness, confusion, slurred speech, agitation and seizures) and a late depressive phase (e.g. coma and respiratory arrest). Cardiovascular toxicity is classically triphasic, including an early phase (e.g. hypertension and tachycardia), an intermediate phase (e.g. myocardial depression and hypotension) and a terminal phase (e.g. arrhythmias and cardiac arrest). The symptoms of central nervous system toxicity generally precede those of cardiovascular system toxicity. However, cardiovascular symptoms may appear suddenly, and severe and fatal manifestations may occur without initial or mild symptoms [4].

Pregnancy is a risk factor for LAST. Pregnant women are particularly vulnerable because of (1) pregnancy-induced hormonal changes in oestradiol and progesterone, which sensitise myocardial cells to arrhythmias and increase neuronal susceptibility to anaesthetics, reducing the seizure threshold; (2) pregnancy-related decreases in protein (alpha-1 acid glycoprotein) titer, which increase free, i.e. toxic, local anaesthetics in the plasma; and (3) epidural venous engorgement caused by expanded blood volume during pregnancy, which increases drug absorption and risk of intravascular epidural catheter migration or placement [2, 5–8]. Local anaesthetics are widely used during labour and birth: over 60% of women in the UK [9], 37–80% in the US [10] and 84% in France [11] receive anaesthetics (e.g. epidural, spinal and combined spinal-epidural anaesthesia for labour and birth). LAST in pregnant women may become more prevalent given the increasing numbers of pregnant women using local anaesthetics, for both labour analgesia and anaesthesia for surgery. Advanced maternal age, obesity and comorbidities such as cardiac disease may increase vulnerability to LAST [2].

Intravenous lipid emulsion is a method for managing LAST. Lipid emulsion comprises an oil-in-water emulsion of soya oil stabilised in egg lecithin and was initially developed for parenteral nutrition in the 1960s [12]. Its role as an antidote for LAST emerged in 1998 when Weinberg et al. [13] observed that the infusion of soybean oil emulsion improved resuscitation rates from severe bupivacaine overdose-induced cardiovascular collapse in rats. After animal studies [14], the first human case report of the successful use of a lipid infusion for resuscitation from a prolonged cardiac arrest after overdose of bupivacaine was published in 2006 [15].

Several mechanisms have been proposed to explain the action of lipid emulsion in LAST [16–19]. The partitioning theory, known as the ‘lipid sink’ theory, is the most widely accepted, proposing that highly lipid-soluble drugs, including local anaesthetics, are absorbed into a lipid emulsion, administered intravascularly, and removed from tissues. This reduces the local anaesthetic concentration in the myocardium [13, 17]. The lipid pharmacokinetics in maternity patients has not yet been elucidated.

Various clinical guidelines [20–25] recommend lipid emulsions for LAST treatment. However, the guidelines do not mention the obstetric population specifically, and the safety of lipid emulsions for pregnant patients, including foetal risk and safe dosages, has not yet been established [2, 26]. Our aim in this scoping review was, therefore, (1) to systematically identify and map the available evidence regarding the efficacy and safety of intravenous lipid emulsion for treating LAST in maternal patients and (2) to identify research gaps in the existing literature.

## Methods and analysis

We conducted the scoping review following the Joanna Briggs Institute Scoping Review Implementation Guidance [27]. For reporting, we followed the Preferred Reporting Items for Systematic Reviews and Meta-Analyses Extension for Scoping Reviews [28].

### Identifying relevant studies

We searched electronic databases – Medline, Embase and Cochrane Central Register Controlled Trials – from January 2000 to Sep 30, 2022, including their reference lists, and reviews for potential additional studies. We developed search strategies for each database using combinations of index terms (e.g. Medical Subject Heading [MeSH]) and free-text terms for ‘pregnancy’, ‘anaesthesia’, ‘resuscitation’ and ‘lipid’. Table 1 displays an example of a search strategy from one bibliographic database (Supporting information: S1 Table). We conducted the search in July 2021 and updated it on September 30, 2022. We also identified maternity cases of LAST reported to the clinical registry, lipidrescue.org [29], developed by Dr. Guy Weinberg, with his permission.

**Table 1:** Maximum recommended doses (for ideal body weight and nonspecific injection sites)

Local Anaesthetic	Maximum dose without epinephrine (mg/kg)	Maximum dose with epinephrine (mg/kg)
Lidocaine	4.5	7
Bupivacaine	2	3
Levobupivacaine	2	3
Ropivacaine	3	3
Chloroprocaine	11	14

University of Iowa health care, 2019

### Study selection

We used the web-based software Covidence to screen and review the papers. At least two reviewers (MN and MT) independently reviewed the title or abstract and full text against inclusion and exclusion criteria. Inclusion criteria were studies of obstetric patients with signs and symptoms of LAST. We included any type of study design containing primary quantitative data, including case reports. We excluded nonhuman studies. No restriction was placed on the year of publication or language. If two reviewers voted differently on whether a study met the inclusion criteria, a third reviewer was involved and discussion was conducted until a consensus was reached.

### Data charting

We created a table to extract the following data from the included studies: author; year of publication; patient characteristics; local anaesthetic type; dose used; signs and symptoms of LAST; treatment administered for LAST; whether lipid emulsion was used, and if so, the dose; and patient outcomes. Data extraction was carried out by MT, MN and MF.

### Result collating, summarising and reporting

We narratively summarised case reports according to the characteristics of the patient with LAST, treatments for LAST with or without intravenous lipid emulsion, and outcomes. In each case, we compared the doses of administered local anaesthetics with the maximum recommended dose for adult patients (Table 1) wherever possible. There are no guidelines nor is there any consensus on the safe doses of anaesthetic for pregnant women, but because pregnant women are thought to tolerate a lower dose than the general adult population, any dose exceeding the maximum dose for the general adult population was considered an overdose.

Similarly, because there are no guidelines on the dosing and timing of lipid emulsion administered for pregnant patients with LAST, the lipid doses used in each case were compared with guidelines developed for the general population [20, 24, 30–32] – e.g., the initial 1.5 mL/kg 20% lipid bolus with the maintenance infusion of 0.25 mL/kg/minute ideal body (Table 2).

**Table 2:** Summaries included studies

**Authors/Year**	**Patients**	**Block type**	**Local Anaesthetic**	**Central nervous system symptoms**	**Cardiovascular symptoms**	**Possible causes**	**Lipid emulsion (20%)**		**Other treatment for LAST**	**Outcomes**
**LAST treated with lipid**										
Anada & Yoshida 2017 [23]	Age: 28 Gestation: 35 wks 150 cm, 50 kg (pre-pregnancy 40 kg)	TAP block after CS with general anaesthesia	Ropivacaine 225 mg	Restlessness Seizure	Hypertension Tachycardia Hypotension Cardiac arrest	Overdose of ropivacaine	Intralipos	Boluses 1.5 ml/kg/min (75 ml) × 3 boluses at 5-min intervals Infusion 0.250.5 ml/kg/min	ACLS including intubation and defibrillation for VF, adrenaline 1 g, midazolam 5 mg	Mother: ROSC, Symptoms resolved Baby: N/A
Diaz et al. 2012 [33]	Age: NR Gestation: Term– cm, 75 kg	CS after epidural for labor	CS: Lidocaine 340 mg Labour: levo-bupivacaine 34.25 mg over 3.5 h	Nausea, Drowsiness, Hand tremors, Nystagmus, Loss of consciousness	Hypotension	NR	Media-lipide	Bolus 100 ml over 5 min Infusion 400 ml	Phenylephrine Ondansetron	Mother: Symptoms resolved Baby: N/A
Dun-Chi Lin et al., 2017 [34]	Age: 29 Gestation: 39 wks 163 cm, 87.3 kg	Combined spinal–epidural for labor + PCEA	Lidocaine 45 mg + epinephrine Bupivacaine 29.25 mg over 50 min	Tinnitus Metallic taste Experience of ‘something was off’ ‘having an out-of-body’	Tachycardia Hypertension Palpitations	Intravascular migration of an epidural catheter	Intralipid	Bolus 1.5 mL/kg. Infusion 0.25 mL/kg/min	Oxygen	Mother: Symptoms resolved Baby born healthy by CS
Naidu & Richebe 2013 [35]	Age: 25 Gestation: 37 wks 150 cm, 51 kg	TAP block after CS with general anaesthesia	TAP: Bupivacaine 150 mg × 2 with 15 h interval CS: lidocaine 60 mg	Seizure	No hemodynamics stable	NR	Intralipid	Bolus 1.5 ml/kg	Intubation, lorazepam, propofol	Mother: Symptoms resolved Baby: N/A
Singh et al 2019 [36]	Age: 28 Gestation: 38 wks – cm, 75 kg	Epidural for labor	Isobaric bupivacaine 20 mg	Agitation Twitching of face or /limbs	Tachycardia Hypertension	NR	Intralipid	Bolus 1.5 ml/kg (112.5 ml) × 2 at 10 min interval	NR	Mother: Symptoms resolved Baby born healthy by CS
Spence et al. 2007 [37]	Age: 18 Gestation: 38 wks – cm, 86 kg	Epidural for labor CS	Labour: lidocaine 80 mg, isobaric bupivacaine 15 mg CS: bupivacaine 50 mg	Restlessness Agitation face/limbs twitching Unresponsiveness Seizure	Tachycardia Hypertension	Intravascular migration of an epidural catheter	Intralipid	Bolus: 100 ml Infusion: 400 ml	Diazepam MgSO4–labetalol infusions	Mother: Symptoms resolved Baby born by CS
Weiss et al. 2014 [38]	Age: 36 Gestation: 38 wks 152 cm, 56 kg	TAP block after CS with spinal anaesthesia	TAP: levo-bupivacaine 150 mg CS: Hyperbaric bupivacaine 10 mg	Unresponsive Seizure Respiratory arrest	None Hemodynamic parameters stable	Partial intramuscular injection	Intralipid	Bolus 100 ml over 2 min Infusion 0.25 ml/kg/min (total: 200 ml)	Bag-mask ventilation Benzodiazepine	Mother: Symptoms resolved Baby: N/A
	Age: 33 Gestation: 36 wks 170 cm, 61 kg	TAP block after CS with intrathecal anaesthesia	TAP: ropivacaine 300 mg CS: hyperbaric bupivacaine 10 mg	Seizure Respiratory arrest	‘No hemodynamic change was noted’	Overdose of ropivacaine	Intralipid	Bolus 100 ml over 2 min. Infusion 0.25 ml/kg/min. (total:250 ml)	Bag-mask ventilation Medication for high blood pressure	Mother: Symptoms resolved Baby: N/A
**Lipidrescue.org (Weinberg) ** [29] Cases 1–7										
Case 1, posted on May 21, 2011	Age: 20 Gestation: NR – cm, 60 kg	Epidural for perineal tear after epidural for labour	Perineal repair: Bupivacaine 50 mg Labor: Bupivacaine 100 mg over 4 h	Loss of consciousness, Seizure	Hypertension (LAST or pre-existing unknown)	Intravascular migration of an epidural catheter	Intralipid	Bolus 100 ml Infusion (rate: NR)	Intubation Thiopentone Suxemethonium Magnesium	Mother: Symptoms resolved Baby: N/A
Case 2, posted on Apr 14, 2013	Age: 40 Gestation: NR – cm, 62 kg	Epidural for labor	Bupivicaine 105 mg	Dizziness, Tingling of nose and cheeks	Tachycardia	NR	Intralipid	Bolus 100 ml over 2–3 min	NR	Mother: Symptoms resolved Baby born healthy by vaginal birth
Case 3, posted on May 1, 2014	Age: NR Gestation: NR – cm, – kg	TAP block after CS with epidural anaesthesia	TAP: Bupivacaine (Marcaine) (150 mg) CS: lidocaine (380 mg)	Hearing and taste abnormalities ‘Difficulty with speech and movement’	Hypertension	NR	Type: NR	Bolus 1.5 ml/kg. Infusion 0.25 ml/kg/min. (total 500 ml)	100% non-rebreather mask	Mother: Symptoms resolved Baby: N/A
Case 4, posted on Jul 17, 2016	Age: 21 Gestation: NR – cm, – kg	N/A	Bupivacaine 250 mg	Perioral numbness, Tinnitus, Seizure, Stupor, Apnoea	Tachycardia Hypotension	Wrong route drug error	Intralipid	Bolus 150 ml. Infusion 0.25 ml/kg/min for 15 min	No CPR	Mother: Symptoms resolved Baby born healthy by vaginal birth
Case 5, posted on Feb 27, 2020	Age: 29 158 cm, 62 kg	Spinal for CS	Bupivacaine 12.5 mg with epinephrine	NR	Arrhythmia	NR	Intralipid	Bolus 70 ml + 25 ml at 3-min intervals	NR	Mother: Symptoms resolved (apart from heartburn) Baby: NR
Case 6, posted on Dec 8, 2020	Age: NR Gestation: 26 wks – cm, – kg	Infiltration anaesthesia for fetal thoracentesis	Lidocaine 800 mg without epinephrine	Short of breath, Dizziness, Slurred speech, Seizure, Loss of consciousness, Respiratory arrest	NR	Overdose of lidocaine	Type: NR	Bolus 100 ml. Infusion 0.25 ml/kg/min for 1 h	Intubation Midazolam Propofol	Mother: Symptoms resolved Baby born alive by CS
Case 7, posted on Jan 3, 2021	Age: 35 Gestation: NR 163 cm, 66 kg	Ilioinguinal nerve block after CS with spinal anaesthesia	Ropivacaine 112.5 mg CS: heavy bupivacain 9 g	Dizziness, Tinnitus, Slurred speech, Breathing difficulties	None ‘ECG and BP, HR remained normal’	NR	Intralipid	Bolus 90 ml	NR	Mother: Symptoms resolved Baby: N/A
LAST treated without lipid										
Griffiths et al. 2013 [39]Cases 1–3	Age: mean = 34 (range 32–36) Weight: mean = 91 (SD = 6.4) BMI: mean = 34.8 (SD = 6.4)	TAP block after CS with spinal anaesthesia	TAP: ropivacaine mean = 229 mg (SD = 19.1)	Case 1. Perioral tingling slurred speech	‘No patients exhibited evidence of cardiovascular disturbance’ (p. 998)	NR	Not used	N/A	‘All symptoms resolved without treatment’ (p.998)	Mother: Symptoms resolved Baby: N/A
				Case 2. Perioral tingling tongue paraesthesia						
				Case 3. Metallic taste						
Ideno et al. 2013 [40]	Age: 20 Gestation: 37 wks 165 cm, 49 kg (prepregnancy 42 kg)	TAP block after CS with spinal anaesthesia	TAP: lidocaine 200 mg Ropivacaine 150 mg CS: hyperbaric bupivacaine 10 mg	Dizziness Dysarthria, Numbness of lips, Visual disturbance	None	NR	Not used	N/A	Colloid infusion	Mother: Symptoms resolved Baby: N/A
Wada et al. 2011 [41]	Age: 28 Gestation: 32 wks	TAP block after CS	Ropivacaine (dose: NR)	Restlessness Seizure	NR	NR	Not used	N/A	Magnesium sulphate diazepam	Mother: Symptoms resolved Baby: N/A
Smetzer et al 2010 [42]	Age: 16	N/A	Intravascular infusion of Bupivacaine	Seizure Respiratory distress Apnoea	Cardiac arrest	Wrong route drug error	Not used	N/A	ACLS	Mother: Death Baby born healthy by CS
Sud & Szawarski 2018 [43]	Age: 30	N/A	Intravascular infusion of bupivacaine 150 mg	Dizziness, Loss of consciousness, Seizure	Ventricular fibrillation Cardiac arrest	Wrong route drug error	Not used	N/A	ACLS	Mother: Death Baby: N/A

*PCEA* Patient-controlled epidural anaesthetic, *BP* Blood presser, *HR* Heart rate, *CS* Caesarean section, *ECG* Electrocardiogram, *ROSC* Return of spontaneous circulation, *TAP* Transversus abdominis plane block, *wks* gestational weeks

## Results

**Fig. 1:**
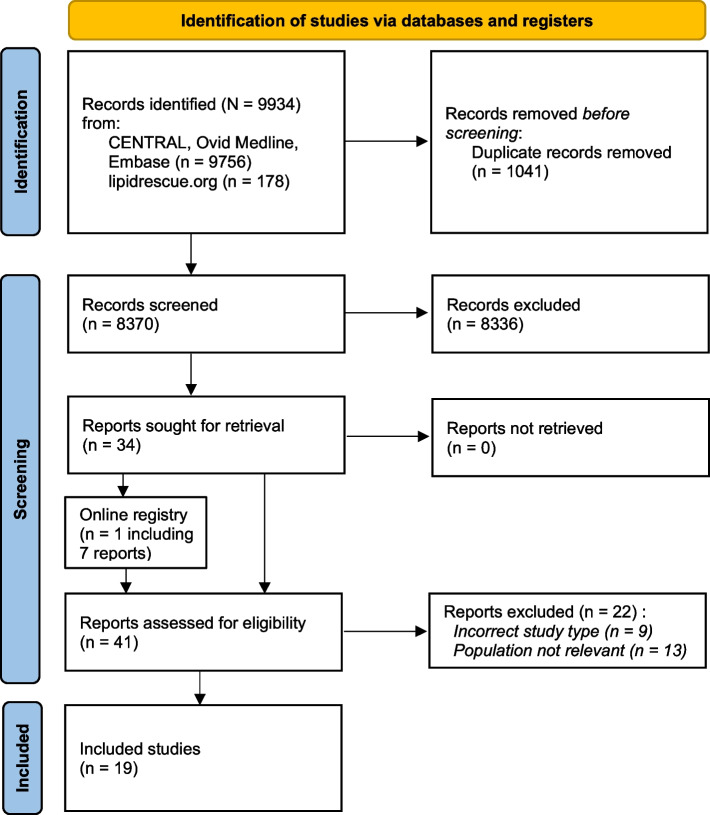
PRISMA flowchart of papers screening process

Our electronic search yielded 8,370 articles after eliminating duplicates. Following the screening of titles and abstracts according to the eligibility criteria, we conducted a full-text screening of 34 articles. We found a further seven cases reported on lipidrescue.org. As a result of the full-text screening (*n* = 41), we included 19 studies (18 case reports or series, and one cohort study), including 22 obstetric patients with LAST. Figure 1 shows the detailed process of the study selection, and Table 2 summarises included studies.

### Characteristics of LAST

#### Patients

Maternal age ranged from 16 to 40 years. Seven women were full-term (≥ 37 weeks’ gestation) [33–38, 40], four were preterm (< 37 weeks’ gestation) [23, 29, 38, 41] and eleven were of unknown gestational age when LAST occurred [23, 29, 39, 42–44]. Six women had pre-existing or pregnancy-induced medical conditions [34, 35, 37, 38, 41]; three were healthy [29]; and medical conditions were not reported for the rest. LAST occurred during pregnancy (*n* = 1), during labour and birth (*n* = 8) or after birth (*n* = 13; Table 3).

**Table 3:** Characteristics of LAST

	Cases, N
**Onset of LAST (*n* = 22)**	
After birth	13
During labour or birth including CS	8
In pregnancy	1
**Types of local anaesthesia (*n* = 19 excluding drug errors)**	
TAP block after CS	11
Epidural CS (preceded by epidural for labour)	2
Epidural for labour	2
Spinal for CS	1
Combined spinal–epidural (CSE) for labor	1
Perineal nerve block	1
Infiltration anaesthesia for fetal thoracentesis	1
**Types of anaesthetic(*n* = 19, numbers overlapping)**	
Bupivacaine	18
*Bupivacaine only*	*(8)*
*Bupivacaine* + *other*	*(10)*
Ropivacaine	9
*Ropivacaine only*	*(2)*
*Ropivacaine* + *other*	*(7)*
Lidocaine	7
*Lidocaine only*	*(1)*
*Lidocaine* + *other*	*(6)*
Levobupivacaine	1
*Levobupivacaine* + *other*	*(1)*
**Potential primary causes of LAST (*n* = 22)**	
Overdose	4
Intravascular migration of an epidural catheter	3
Partial intramuscular injection	1
Wrong route drug error	3
NR or unclear	11
**Clinical manifestations (*n* = 22)**	
Central nervous system toxicity	
*Early phase*	9
*Severe phase (seizure, loss of consciousness)*	12
*Unclear*	1
Cardiovascular symptoms	
*No*	8
*Early phase*	5
*Severe phase without cardiac arrest phase*	3
*Cardiac arrest*	3
*Unclear*	3
**Timing of lipid emulsion (*n* = 15)**	
**Early phase of LAST**	5
**Later/severe phase of LAST without cardiac arrest**	9
**Later/severe phase of LAST with cardiac arrest**	1
**Lipid dosage (*n* = 15)**	
1 bolus only without any infusion	3
1 bolus and infusion^a^	9
2 boluses without any infusion	2
2 bolus and infusion	1

^a^This category includes Spence [37] who reported 100 ml of 20% intralipid, provided by two 50 ml boluses

#### Types of blocks, and anaesthetics

Anaesthetic procedures causing LAST, in order of prevalence, included bilateral transverse abdominal plane (TAP) block (*n* = 11) [23, 29, 35, 38–41], epidural top-up anaesthesia for caesarean section (*n* = 2) [33, 37], epidural analgesia for labour (*n* = 2) [29, 36], spinal for caesarean section (*n* = 1) [29], combined spinal–epidural for labour (*n* = 1) [34], perineal nerve block for third-degree tear (*n* = 1) [29] and infiltration anaesthesia for foetal thoracentesis (*n* = 1). In series of procedures during pregnancy, labour and birth, and immediately after birth, the most commonly reported anaesthetic drug used was bupivacaine (*n* = 18), followed by ropivacaine (*n* = 9); these were administered alone, in combination with others, or preceding or following another local anaesthetic drug.

#### Potential causes of LAST

Of the causes reported (*n* = 10), the most frequently cited was drug overdose (*n* = 3) [23, 29, 38], Although not reported by the original authors, we identified one more case of an overdose in which the patient received a local anaesthetic dose exceeding the maximum recommended dose for an adult [40]. Both intravascular migration of the epidural catheter [29, 34, 37], and wrong route drug errors [infusions containing bupivacaine were accidentally connected to peripheral venous lines] (*n* = 3) [29, 42–44], were mentioned in 3 reports each, followed by unintentional partial intramuscular injections of a local anaesthetic (*n* = 1) [38]. In most cases, assessing drug overdosage was impossible because of the lack of information about the patient’s weight, the use of epinephrine or adrenaline (which reduces systemic absorption and maximum plasma concentrations of a local anaesthetic) [23, 29, 35–38, 40, 42, 43] or dose of local anaesthetic used [41].

#### Clinical manifestations

All cases but one reported signs of neurologic toxicity; of these, 12 patients progressed to severe symptoms including seizures, loss of consciousness, apnoea and respiratory arrest [23, 29, 33, 35, 37, 38, 41–44] with seizure the most frequently reported (*n* = 11) [23, 29, 35, 37, 38, 41–44]. Eleven patients experienced cardiovascular toxicity symptoms [23, 29, 33, 34, 36, 37, 43, 44]. Of these, the most commonly reported were tachycardia (*n* = 6) [23, 29, 34, 36, 37] and hypertension (*n* = 5) [23, 29, 34, 36, 37]. Symptoms of serious cardiovascular toxicity, including hypotension and arrythmia, were present in six patients [23, 29, 33, 42–44], three of whom had cardiac arrest [23, 42–44].

### Intravenous lipid emulsion and other treatment for LAST

Intravenous lipid emulsion was administered in 15 cases but were not reported or were not administered in 7 cases. Although data were often incomplete, of the 15 cases of lipid administration, this was the sole treatment in four cases with mild symptoms [29, 34, 36], in addition one patient also received 100% oxygen from a non-rebreathing mask [29]. Of the nine patients who were given lipids for severe LAST symptoms without cardiac arrest, seven received concurrent airway management [29, 35, 37, 38], and of these seven, five also had anticonvulsants [29, 35, 37, 38]. In the remaining cases, there was no information other than the HDU admission [29] or the report of no need for CPR [29]. One patient who went into cardiac arrest during TAP block after a caesarean section had intravenous lipid emulsion along with seizure management and advanced cardiovascular life support [23]. All patients who developed LAST before delivery, including during pregnancy, ended up with a caesarean birth [29, 33, 34, 36], except one [29].All intravenous lipid emulsions used were 20% concentration, and most of them were intralipid (*n* = 12). Of the seven cases without lipid emulsion administration, two occurred in 2006 (the year the first human study of intravenous lipid emulsion for LAST was published [15]) or earlier.

#### Timing of intravenous lipid emulsion administration

The timing of lipid emulsion administration varied. In five reported cases, a lipid emulsion was started at the onset of the early neurologic symptoms (dizziness, agitation, or twitching of face or limbs) or early cardiovascular symptoms (hypertension or tachycardia), before severe symptoms of LAST developed [29, 34, 36]. In 10 cases, lipid emulsion was delayed until the onset of more severe symptoms (i.e. seizure, loss of consciousness, respiratory arrest, hypotension or arrythmia) but without cardiac arrest [29, 33, 35, 37, 38, 41]. In one case, lipid emulsion was administered once cardiac arrest occurred [23].

#### Dosage and rates of intravenous lipid emulsion administration

Of the five patients [29, 34, 36] who received 20% lipid emulsion in an early phase of LAST, all received an initial bolus, but the dosage and rate of the bolus and administration of the infusion varied. For example, only two of these patients received an infusion after the initial bolus [29, 34]. Of the three patients who received a bolus only, one [29] received a dose of 90 ml ‘slowly’ (a slightly lower dose [1.4 ml/kg] than the recommendation), one had a bolus of 100 ml over 2–3 min [29], and the other had two boluses of 1.5 ml/kg (112.5 ml) at 10-min intervals [36].

Of the nine patients who suffered severe LAST (seizure, loss of consciousness, respiratory arrest, hypotension or arrhythmia) but not cardiac arrest, seven received a bolus of intravenous lipid emulsion followed by an infusion [29, 33, 37, 38], one received a bolus only, and another received two boluses without infusion. Four of these patients with severe LAST received higher bolus doses than recommended by the guidelines: three patients weighing 56–61 kg received a 100 ml lipid bolus (equivalent to 1.6–1.8 ml/kg) [29, 38] and another patient of unknown weight (who had a direct intravenous injection of bupivacaine) received a 150 ml lipid bolus. Information about the rate of lipid emulsion administration was only available in three cases involving a bolus (i.e., over 2 min [38] or 5 min [33]) and in five cases involving infusion (i.e. 0.25 ml/kg/min [29, 38] and 400 ml for a 75 kg patient over 2 h [equivalent to 0.04 ml/kg/min [33]). Among severe cases without cardiac arrest, the symptoms improved after a single bolus in all except one case where a second smaller bolus was administered 3 min later [29].

One patient in cardiac arrest was given an initial bolus of 20% lipid emulsion at 1.5 ml/kg/min and an infusion at 0.25 ml/kg/min [23]. With no return of spontaneous circulation after 5 min, the infusion rate was doubled to 0.5 ml/kg/min, and two additional lipid boluses were given at 5-min intervals [23]. This is in line with the dosage and rate recommended in the AAGBI guidelines [20], as assessed by the current reviewers against existing guidelines [20–22, 24, 25].

#### Treatment without intravenous lipid emulsion

Of the five cases of the patients with mild toxicity, three received no treatment [39]: one received a colloid infusion (500 ml; a lipid was prepared but not administered as symptoms improved) [40]. Another patient received anticonvulsants at the onset of seizure with no lipid therapy. In two publicly reported cases of medication errors involving intravascular injection of bupivacaine, cardiovascular collapse occurred necessitating advanced life support [42–44]. One of these cases occurred after vaginal birth [43, 44] and the other during labour, resulting in an emergency caesarean section [42].

### Outcomes

Symptoms of LAST resolved in all women treated with an intravenous lipid emulsion. More specifically, one case report described a woman in cardiac arrest in whom return of spontaneous circulation occurred 13 min after cardiac arrest following advanced life support and three boluses of lipid emulsion. In five women without cardiac arrest, symptoms of severe neurological or cardiovascular toxicity improved rapidly following intravenous lipid administration (eg. ‘within 30 s’ [37, p 517], within approximately 2–3 min [29, 33], and within ‘five minutes’ [34p, 248] of initiating lipid therapy), whereas for the remaining four patients, the time for symptom resolution was unclear [29, 35]. In five women with mild toxicity treated with lipid, symptoms became stable immediately [29], within 10 min [34] and within 20 min [36] after intralipid emulsion administration, but the time of resolution was unclear in two cases [29].

Of the seven patients who did not receive lipid administration [39–44], five women with LAST after TAP block recovered without neurological sequelae [39–41]; one recovered from symptoms after 3 h [40], and the remaining four [41], the time to recovery was not stated. Another two women who did not receive lipids for intravenous bupivacaine-induced cardiac arrest died despite resuscitation efforts [42–44]. None of the cases reported adverse events following the lipid emulsion administration.

Where fetal outcome was reported, all babies born at 37 or more weeks gestation, to women who suffered LAST prior to delivery survived. [34, 36, 37] No details were reported on a baby born at 26 weeks’ gestation except that the baby was born alive [29].

## Discussion

In this scoping review, we sought to systematically identify and map the evidence on the efficacy and safety of intravenous lipid emulsions for the treatment of LAST in obstetric patients. When given, lipid emulsion appeared to be effective in all cases although publication bias is likely. Notably, no adverse events due to intravenous administration of lipid emulsion were reported in any cases in either mothers or neonates, even though dosing guidelines were sometimes not followed.

### Comparison with existing studies and guidelines

#### Characteristics of LAST

Previous reviews in the general population have determined that most LAST events are due to increased sensitivity to anaesthetic agents rather than drug overdose [6]. During pregnancy, physiological changes may increase sensitivity to local anaesthetics and thus exceed *minimum* toxic plasma concentrations even when the recommended maximum tolerated dose of local anaesthetics is adhered to [2, 39, 45]. Excluding the cases of wrong-route drug error, at least one-third of the women had pregnancy-induced complications (e.g., preeclampsia or acute fatty liver of pregnancy) or preexisting medical conditions (e.g., hypertension, type 1 diabetes, congenital renal malformation, or obesity), which could have contributed to the risk for toxicity.

However, overdose was still the most commonly reported LAST cause in our review, most commonly occurring in TAP block associated with general or spinal anaesthesia for caesarean section. The potential danger of TAP blocks in these situations has not previously been highlighted. TAP blocks involve the injection of a large volume of local anaesthetic into a relatively vascular plane [39, 46], which may result in significant local anaesthetic absorption [47], leading to higher concentrations in the blood.

Another point that might warrant attention is the number of cases where a combination of local anaesthetics was used. In several cases the dose of each individual drug did not exceed the recommended maximum, but there appeared to be no consideration of additive effects. Conversion of epidural analgesia for labour to epidural extension (from epidural anaesthesia during labour to surgical anaesthesia for caesarean section may increase the risk of toxicity because of the large doses of d local anaesthetics used. [2, 48, 49]. In several cases the low body weight of the patient seems to have been ignored when the dose of local anaesthetic was determined. Intravascular migration of epidural catheters was also commonly cited as the cause of LAST and appeared to be associated with patient mobilisation and epidural catheters that had been in-situ for many hours in labour.

A smaller proportion of cases suffered from seizures than reported in the non-pregnant population, although this was the most common symptom [50, 51]. In contrast, prodromal symptoms (e.g., dizziness, confusion, tinnitus, and slurring) were reported more frequently in cases in the obstetric population [50, 51]. Vasques et al. [50] argued that the increase in reporting of prodromal symptoms reflects increased awareness of early detection and diagnosis of LAST following the publication of clinical guidelines for LAST management [20, 21, 52]. The current review seems to reflect this trend.

#### Intravenous lipid emulsion

The timing of the initiation of lipid emulsion in LAST is controversial. In early case reports in 2006 and 2008 [15, 53, 54], lipid was administered to patients only when standard advanced cardiovascular life support did not achieve return of spontaneous circulation. The 2010 ASRA advisory on LAST suggested the use of lipid emulsion for patients suffering local anaesthetic-induced arrhythmia, prolonged seizures or rapid clinical deterioration [21]. More recently, a growing number of case reports suggest the benefit of prompt administration of lipid emulsion as a first-line treatment along with CPR.

In our review of obstetric patients, one patient with cardiac arrest received intravenous lipid emulsion according to clinical guidelines [20, 30]. However, liberal use was often found for patients not in cardiac arrest. Although under and overdosing was common, there were no adverse sequlae reported. In an emergency, calculating weight-based dosing may hinder timely lipid administration [55]. The recent ASRA practice advisory on LAST (in the 2017 and 2020 versions) simplified the instructions for administering lipid emulsion, particularly for patients over 70 kg, in whom a weight based dose is no longer recommended (i.e., a 100 ml lipid bolus and infusion of 200–250 ml) [31]. In our review clinicians appeared to prioritise timely lipid administration over the precise calculation of doses during a LAST crisis.

#### Outcomes

There was a notable difference in clinical outcomes in three cases where anaesthetics were mistakenly administered intravenously; one woman receiving lipids with conventional resuscitation survived [29], and two women died with conventional resuscitation alone [42, 43]. Because these results are based on case reports and there is no comparison group from the same population, it is impossible to assess whether the favourable outcome resulted from lipid emulsion administration.

We did not find any case reports of obstetric patients where the use of lipid emulsifiers failed to resolve LAST symptoms or where it caused adverse events. A review of non-obstetric patients indicates pancreatitis can occur as a result of use of intravenous lipid emulsion therapy [56]. A systematic review of clinical adverse events after acute intravenous lipid emulsion administration included acute kidney injury and acute lung injury [12]. Pregnant women with severe hyperemesis gravidarum treated with total parenteral nutrition may be at risk of uterine contractions with a high lipid infusion rate [57, 58]. It is important to be cautious about lipids’ potential adverse effects [12]. Further research is needed to accumulate data, including adverse events, to facilitate evidence-based decision-making and clinical practice.

### Strengths and limitations

The administration of lipid emulsions for the treatment of LAST, is widely advocated but based on limited data especially with respect to the obstetric population [59–61]. Our review collated more cases of pregnant women than any existing review.

One important limitation of this review is that results showing the beneficial effects of intravenous lipid emulsion are based on studies without comparison groups. Although case reports are a valuable source of clinical information [62], these reports are highly individual and heterogeneous. Some of the cases reported were not peer reviewed, and treatment details other than lipids were sometimes incomplete.

In view of our search strategy, case reports of LAST treated without lipid may be missing if the word *lipid* or *fat* is not included. Furthermore, the results of case reports were subject to publication bias because cases with strong positive results tend to be highlighted and published.

Further case reports and observational studies detailing clinical settings and personal backgrounds may be required on an ongoing basis to identify characteristics of patients who may benefit or experience harm from lipids.

## Conclusion

With the increase of caesarean sections worldwide, the management and treatment of LAST in pregnant women is an increasingly important issue [63]. Anaesthetists use local anaesthesia more often than general anaesthesia in pregnant women [30]. However, physiological changes that make regional anaesthesia safer in pregnancy also increase the risk of LAST [12]. In this scoping review, we attempted to identify and map the available evidence regarding the efficacy and safety of lipid emulsions to treat LAST in pregnant women. It will be necessary to accumulate data on LAST and its management because this would allow comparisons to be made on the clinical outcomes of different methods for LAST management. Such a global database would enable clinicians to conduct a risk–benefit analysis of lipids and to facilitate evidence-based decision-making for clinical practice.

### Supplementary Information

**Additional file 1: S1 Table.** Search strategy (Medline OvidSP) 1970 to September week 2, 2022.

**Additional file 2: S2 Table.** Guidelines on lipid emulsion therapy for LAST.

## Data Availability

The datasets acquired and/or analysed during the current study are available from the corresponding author on reasonable request.

## Abbreviations

LAST: Local anaesthetic systemic toxicity
AAGBI: The Association of Anaesthetists of Great Britain and Ireland
ASRA: The American Society for Regional Anaesthesia and Pain Management
AHA: The American Heart Association
MeSH: Medical Subject Heading
RCT: Randomised control trial
PCEA: Patient-controlled epidural anaesthetic
BP: Blood presser
HR: Heart rate
CS: Caesarean section
ECG: Electrocardiogram
ROSC: Return of spontaneous circulation
TAP: Transversus abdominis plane block
CSE: Combined spinal–epidural
