# Supplementary material for: Intravenous lipid emulsion for local anaesthetic systemic toxicity in pregnant women: a scoping review
Source: BMC Pregnancy Childbirth. 2024 Feb 14;24:138. doi: 10.1186/s12884-024-06309-1 (PMC10865663; doi:10.1186/s12884-024-06309-1)
Supplement: Supplementary file 1 — Additional file 1: S1 Table. Search strategy (Medline OvidSP) 1970 to September week 2, 2022. [file 12884_2024_6309_MOESM1_ESM.docx]

**S1 Table. Search strategy (Medline OvidSP) 1970 to September week 2, 2022**

| 1 | exp Pregnancy Complications, Cardiovascular/ or exp Pregnancy/ or exp Pregnancy, High-Risk/ or exp Pregnancy Complications/ |
| --- | --- |
| 2 | exp Pregnant Women/ |
| 3 | pregnan*.mp. |
| 4 | matern*.mp. |
| 5 | exp Maternal Mortality/ or exp Maternal Death/ |
| 6 | (maternal adj3 morbidit*).mp. |
| 7 | exp Obstetrics/ |
| 8 | obstetric*.mp. |
| 9 | Pregnant wom#n.mp. |
| 10 | parturient.mp. or exp Labor, Obstetric/ or exp Anesthesia, Obstetrical/ |
| 11 | peripartum.mp. or exp Peripartum Period/ |
| 12 | exp Perinatology/ |
| 13 | Perinatal.mp. |
| 14 | gestation*.mp. |
| 15 | gravid*.mp. |
| 16 | matern*.mp. |
| 17 | 1 or 2 or 3 or 4 or 5 or 6 or 7 or 8 or 9 or 10 or 11 or 12 or 13 or 14 or 15 or 16 |
| 18 | exp Heart Arrest/ |
| 19 | (heart adj5 arrest?).mp. |
| 20 | (cardiac adj5 arrest?).mp. |
| 21 | (cardiopulmonary adj5 arrest?).mp. |
| 22 | (cardiovascular adj5 arrest?).mp. |
| 23 | asystole?.mp. |
| 24 | pulseless electrical activit*.mp. |
| 25 | exp Cardiopulmonary Resuscitation/ |
| 26 | exp Resuscitation/ or exp Out-of-Hospital Cardiac Arrest/ |
| 27 | CPR.mp. |
| 28 | resuscita*.mp. |
| 29 | (heart adj3 compression?).mp. |
| 30 | (cardiac adj3 compression?).mp. |
| 31 | (chest adj3 compression?).mp. |
| 32 | (thoracic adj3 compression?).mp. |
| 33 | exp Heart Massage/ |
| 34 | (heart adj3 massage?).mp. |
| 35 | (cardiac adj3 massage?).mp. |
| 36 | (heart adj3 failure?).mp. |
| 37 | (cardiac adj3 failure?).mp. |
| 38 | (cardiovascular adj3 failure?).mp. |
| 39 | (cardiopulmonary adj3 failure?).mp. |
| 40 | (cardiac adj3 collapse?).mp. |
| 41 | (cardiovascular adj3 collapse?).mp. |
| 42 | (cardiopulmonary adj3 collapse?).mp. |
| 43 | cardiovascular.mp. or exp Cardiovascular Diseases/ |
| 44 | cardiac toxicity.mp. or exp Cardiotoxicity/ |
| 45 | peri-arrest state?.mp. |
| 46 | (life adj3 support*).mp. |
| 47 | emergency.mp. or exp Emergencies/ or exp Emergency Medical Services/ |
| 48 | exp Ventricular Fibrillation/ |
| 49 | electromechanical dissociation*.mp. |
| 50 | 18 or 19 or 20 or 21 or 22 or 23 or 24 or 25 or 26 or 27 or 28 or 29 or 30 or 31 or 32 or 33 or 34 or 35 or 36 or 37 or 38 or 39 or 40 or 41 or 42 or 43 or 44 or 45 or 46 or 47 or 48 or 49 |
| 51 | exp Anesthesia/ or exp Anesthesia, Local/ or exp Anesthetics/ or exp Analgesia/ or exp regional anesthesia/ |
| 52 | an?esthe*.mp. |
| 53 | exp Administration, Topical/ or topical.mp. |
| 54 | intoxic*.mp. |
| 55 | toxin*.mp. |
| 56 | toxic*.mp. |
| 57 | exp "Drug-Related Side Effects and Adverse Reactions"/ |
| 58 | exp Pharmacokinetics/ or pharmacokineti*.mp. |
| 59 | exp Bupivacaine/ |
| 60 | bupivacain*.mp. |
| 61 | exp Ropivacaine/ |
| 62 | ropivacaine*.mp. |
| 63 | exp Lidocaine/ |
| 64 | lidocaine*.mp. |
| 65 | exp Prilocaine/ or exp Lidocaine, Prilocaine Drug Combination/ |
| 66 | prilocain*.mp. |
| 67 | exp Mepivacaine/ |
| 68 | mepivacain*.mp. |
| 69 | exp Hypothermia/ or exp Hypothermia, Induced/ |
| 70 | 51 or 52 or 53 or 54 or 55 or 56 or 57 or 58 or 59 or 60 or 61 or 62 or 63 or 64 or 65 or 66 or 67 or 68 or 69 |
| 71 | exp Fat Emulsions, Intravenous/ |
| 72 | lipid.mp. |
| 73 | (fat adj3 emulsion*).mp. |
| 74 | (fat adj3 resuscitation*).mp. |
| 75 | 71 or 72 or 73 or 74 |
| 76 | 17 and (50 or 70) and 75 |
| 77 | limit 76 to humans |
