# Supplementary material for: Intravenous lipid emulsion for local anaesthetic systemic toxicity in pregnant women: a scoping review
Source: BMC Pregnancy Childbirth. 2024 Feb 14;24:138. doi: 10.1186/s12884-024-06309-1 (PMC10865663; doi:10.1186/s12884-024-06309-1)
Supplement: Supplementary file 2 — Additional file 2: S2 Table. Guidelines on lipid emulsion therapy for LAST. [file 12884_2024_6309_MOESM2_ESM.docx]

**S2 Table. Guidelines on lipid emulsion therapy for LAST**

|  | **Initial 20% lipid emulsion** | | | | **Additional lipid** | | | **Max** |
| --- | --- | --- | --- | --- | --- | --- | --- | --- |
|  | Timing of initiation | Dosage of bolus | Bolus Rate | Infusion rate | Timing of additional lipid | Re-bolus | Infusion rate |  |
| ASRA  2020 [22] | ‘Consider administering lipid emulsion early’ (Neal 2021, p.81) | >=70kg:  100 ml  <70kg:  1.5 ml/kg | 2-3 min | >=70kg:  200-250 ml/  13–20 min  <70kg:  0.25 ml/kg/min | If patient remains unstable | <=2 | double rate | 12 ml/kg |
| AAGBI 2010 [20] | In circulatory arrest, give intravenous lipid emulsion.  Without circulatory arrest, consider intravenous lipid emulsion. | 1.5 ml/kg | 1 min | 15 ml/kg/h  (0.25 ml/kg/min) | If cardiovascular stability has not been restored or if an adequate circulation deteriorates | <=2 | double rate | 12 ml/kg |
| AHA 2015^†^ [24] | ‘It may be reasonable to administer ILE, concomitant with standard resuscitative care, to patients with local anaesthetic systemic toxicity and particularly to patients who have premonitory neurotoxicity or cardiac arrest due to bupivacaine toxicity (Class IIb, LOE C-EO)’ (Neumar et al. 2015, S351). | 1.5 ml/kg | 1 min | 0.25 ml/kg/min for 30 to 60 min. | for persistent cardiovascular collapse | <=2 |  | 10 ml/kg over the first hour |
| ERS 2021 [30] | -- | 1.5ml/kg | 1 min | 0.25 ml/kg/min for up to 60 min. | If ROSC has not been achieved at 5 min | <=2 | double rate | 12 ml/kg |
| JSA 2017 [32] | With severe hypotension or arrhythmia, consider intravenous lipid emulsion. | 1.5ml/kg | 1 min | 0.25ml/kg/min | If no improvement in circulation is obtained | <=2 | double rate | 12 ml/kg |

ASRA: American Society of Regional Anesthesia; AAGBI: Association of Anaesthetists of Great Britain and Ireland; AHA: American Heart Association;

ERS: European Resuscitation Council; JSA: Japanese Society of Anesthesiologists

† AHA 2015: Class (strength) of recommendation = Class IIb (weak); Level (quality) of evidence (LOE) = C-EO (expert opinion). The dosage and rate of lipid was from ‘evidence summary’.
